# Author Correction: Antibodies from dengue patients with prior exposure to Japanese encephalitis virus are broadly neutralizing against Zika virus

**DOI:** 10.1038/s42003-024-05963-7

**Published:** 2024-03-01

**Authors:** Gielenny M. Salem, Jedhan Ucat Galula, Shang-Rung Wu, Jyung-Hurng Liu, Yen-Hsu Chen, Wen-Hung Wang, Sheng-Fan Wang, Cheng-Sheng Song, Fan-Chi Chen, Adrian B. Abarientos, Guan-Wen Chen, Cheng-I Wang, Day-Yu Chao

**Affiliations:** 1 Graduate Institute of Microbiology and Public Health, College of Veterinary Medicine, National Chung Hsing University, Taichung City, 402 Taiwan; 2 Institute of Oral Medicine, School of Dentistry, College of Medicine, National Cheng Kung University, Tainan City, 701 Taiwan; 3 Institute of Basic Medical Sciences, College of Medicine, National Cheng Kung University, Tainan City, 701 Taiwan; 4 Graduate Institute of Genomics and Bioinformatics, College of Life Sciences, National Chung Hsing University, Taichung City, 40227 Taiwan; 5 School of Medicine, College of Medicine, National Sun Yat-Sen University, Kaohsiung City, 80424 Taiwan; 6 Center for Tropical Medicine and Infectious Disease Research, Kaohsiung Medical University, Kaohsiung City, 80708 Taiwan; 7 Division of Infectious Diseases, Department of Internal Medicine, Kaohsiung Medical University Hospital, Kaohsiung Medical University, Kaohsiung City, 80708 Taiwan; 8 Department of Medical Laboratory Science and Biotechnology, Kaohsiung Medical University, Kaohsiung City, 80708 Taiwan; 9 Doctoral Program in Microbial Genomics, National Chung Hsing University and Academia Sinica, Taichung City, 402 Taiwan; 10 Singapore Immunology Network, Agency for Science, Technology and Research (A*STAR), 8A Biomedical Grove, Immunos, Singapore, 138648 Singapore; 11 Department of Post-Baccalaureate Medicine, College of Medicine, National Chung Hsing University, Taichung City, 402 Taiwan

**Keywords:** Viral infection, Molecular medicine, Antibodies

Correction to: *Communications Biology* 10.1038/s42003-023-05661-w, published online 24 January 2024

In this article the author name Cheng-I Wang was incorrectly written as Cheng-Yi Wang. The original article has been corrected.

