# Supplementary material for: Antibodies from dengue patients with prior exposure to Japanese encephalitis virus are broadly neutralizing against Zika virus
Source: Commun Biol. 2024 Jan 24;7:15. doi: 10.1038/s42003-023-05661-w (PMC10808242; doi:10.1038/s42003-023-05661-w)
Supplement: Supplementary file 2 — Supplementary Information [file 42003_2023_5661_MOESM2_ESM.pdf]

SUPPLEMENTARY INFORMATION

Antibodies from dengue patients with prior exposure to Japanese encephalitis virus are broadly neutralizing against Zika virus

Gielenny M. Salem[1#], Jedhan Ucat Galula[1#], Shang-Rung Wu[2,3&], Jyung-Hurng Liu[4&], Yen-Hsu Chen[5,6,7], Wen-Hung Wang[5,6,7], Sheng-Fan Wang[6,8], Cheng-Sheng Song[1], Fan-Chi Chen[9], Adrian B.Abarientos[1], Guan-Wen Chen[2], Cheng-I Wang[10], and Day-Yu Chao[1,9,11,*]

[1]Graduate Institute of Microbiology and Public Health, College of Veterinary Medicine, National Chung Hsing University, Taichung City 402, Taiwan

[2]Institute of Oral Medicine, College of Medicine, National Cheng Kung University, Tainan City 701, Taiwan

[3]Institute of Basic Medical Sciences, College of Medicine, National Cheng Kung University, Tainan City 701, Taiwan

[4]Institute of Genomics and Bioinformatics, College of Life Sciences, National Chung Hsing University, Taichung City 402, Taiwan

[5]School of Medicine, College of Medicine, National Sun Yat-Sen University, Kaohsiung City 804201, Taiwan

[6]Center for Tropical Medicine and Infectious Disease Research, Kaohsiung Medical University, Kaohsiung City 80708, Taiwan

[7]Division of Infectious Diseases, Department of Internal Medicine, Kaohsiung Medical University Hospital, Kaohsiung Medical University, Kaohsiung City 80708, Taiwan

[8]Department of Medical Laboratory Science and Biotechnology, Kaohsiung Medical University, Kaohsiung City 80708, Taiwan

[9]Doctoral Program in Microbial Genomics, National Chung Hsing University and Academia Sinica, Taichung City 402, Taiwan

[10]Singapore Immunology Network, Agency for Science, Technology and Research (A*STAR), 8A Biomedical Grove, Immunos, Singapore 138648, Singapore

[11]Department of Post-Baccalaureate Medicine, College of Medicine, National Chung Hsing University, Taichung City 402, Taiwan

*Correspondence: dychao@nchu.edu.tw

#These authors contributed equally to this work.

&These authors contributed equally to this work.

Supplementary Figures: 14

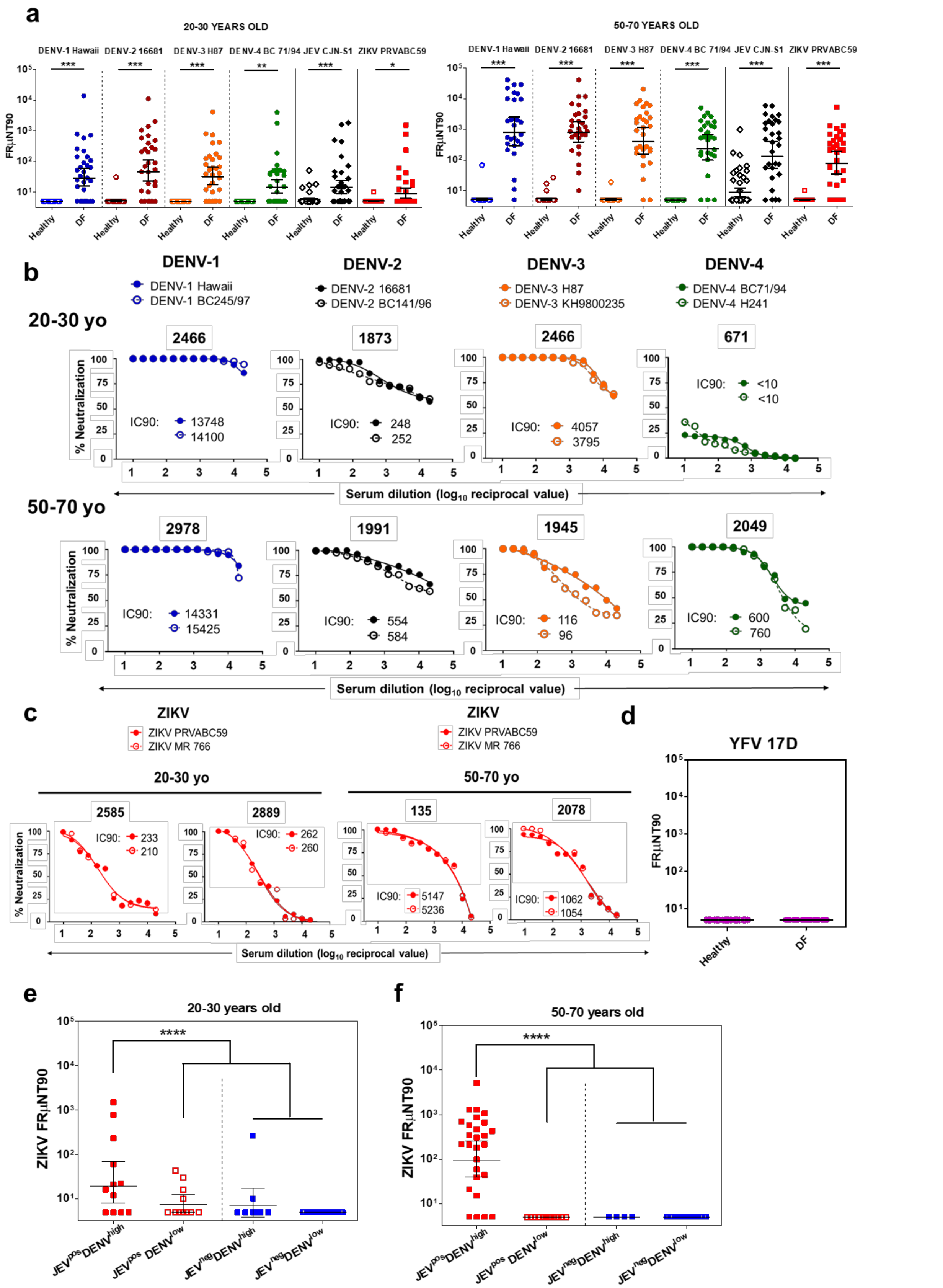

Supplementary Fig. 1: Neutralization profile and assessment of ZIKV immunity among naïve and dengue-immune donors and with age classification.

a, Neutralization antibody profile against DENV-1 to 4 serotypes, JEV, and ZIKV among healthy (n=80) and dengue-infected febrile (DF, n=60) individuals showing the sub-classification according to two age

groups, (a, left) 20-30 years old and (a, right) 50-70 years old. The two age classifications comprised an equal number of healthy (n=40 per group) and DF individuals (n=30 per group). Two-fold serially-diluted plasma samples from healthy and dengue-infected individuals were evaluated for neutralization against the prototype virus strains of DENV-1, DENV-2, DENV-3, DENV-4, JEV, and ZIKV using FRμNT. Each point shows the mean of data from two independent experiments. Shaded and empty circles represent the FRμNT90 values of the 20-30 years old and 50-70 years old groups, respectively. ***, *P*<0.0001; **, *P*<0.001; *, *P*<0.01. **b-d**, Representative neutralization antibody profiles of donor volunteers against the DENV1-4 strains **(b)** and Asian or African ZIKV prototype strains **(c)**, and Yellow fever virus (YFV) **(d)**, including their sub-classification according to two age groups: 20-30 **(b, upper panel)** and 50-70 years old **(b, lower panel)**. Shaded and empty circles represent the FRμNT90 values against the prototype and the recent global or Taiwan clinical isolates, respectively. Each point shows the mean of data from two independent experiments. A nonlinear curve was generated for all antibody dilution series of each donor for all of the assays in **1b** and **1c**. **e,f**, Distribution and statistical comparison of the breadth of ZIKV neutralization among the 20-30-year-old **(e)** and 50-70-year-old **(f)** donors, which were further dichotomized according to the presence (pos) or absence (neg) of protective or high neutralizing FRμNT90 titers against JEV, and high or low neutralizing titers against DENV. In Fig. **1a**, **1e**, and **1f**, the black solid horizontal bars represent the geometric mean titers (GMT) of samples with SD analyzed within each group. FRμNT90 titer <10 was represented with 5 for graphic display and statistical analysis. Significant differences were tested using one-way ANOVA followed by Tukey's multiple comparisons post-test, with levels of significance defined by: ***, *P*<0.0001; **, *P*<0.001. DENV=dengue virus; FRμNT=focus reduction microneutralization test.

a

Donor ID	Sex and Age (y)	Plasma sample	Post-infection collection time	IC90*					
				DENV-1	DENV-2	DENV-3	DENV-4	JEV	ZIKV
KH1891	Female, 68	Early Convalescent	< 1 month	9595	1895	2155	1205	10	338
		Late Convalescent	> 18 months	5453	349	479	44	34	155

DENV, dengue virus; JEV, Japanese encephalitis virus; ZIKV, Zika virus; IC90, 90% inhibitory concentration; ID, identification.

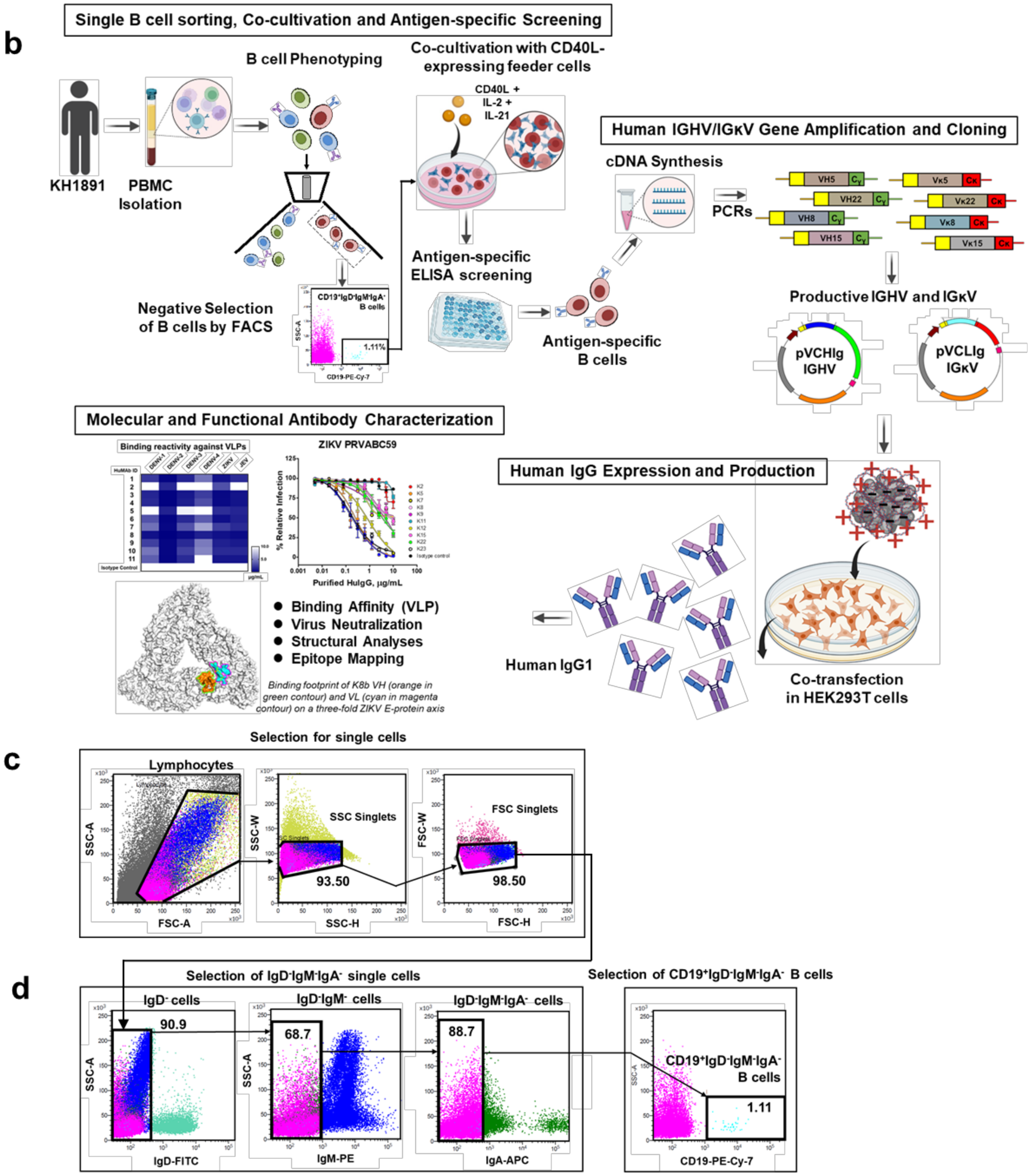

Supplementary Fig. 2: Flow cytometry gating strategy to identify CD19$^+$IgD$^-$IgM$^-$IgA$^-$ single B cells from the PBMCs of a dengue-immune donor, KH1891.

a, Neutralizing antibody titers of donor KH1891 against the prototype strains of the dengue virus serotypes 1 to -4, JEV, and ZIKV at less than one month and 18 months post-infection plasma collection, representing the early and late convalescent stages, respectively. **b**, The pipeline for the discovery and generation of human monoclonal antibodies using the single B-cell technology. From immune donors, PBMCs were prepared for the isolation of monocytes by flow cytometry using a negative selection strategy. The bivariate plot showing the fluorescently labeled, single-sorted human B cells was generated by a licensed software (BD FACSChorus™, v.1.1.18.0). Human B cells were co-cultivated with CD40L-expressing thymoma cells and growth factors for two weeks and screened for antigen specificity using ELISA. Following RT-PCRs, productive human immunoglobulin (Ig) V_H and V_K segments were cloned into in-house generated expression vectors and co-transfected into HEK293T cells for recombinant antibody expression. The plasmid maps were generated using an open-source plasmid visualization software, Plasm (v.2.0). Purified antibodies were tested for VLP binding and live-virus neutralization, followed by epitope mapping and structural data analyses. The representative heatmap of ELISA binding reactivity and % relative infection curves were generated using GraphPad Prism (v.9.5.1, GraphPad Software, LLC), while the binding footprint of K8b with its heavy (orange in green contour) and light (cyan in magenta contour) chains on a three-fold ZIKV E-protein axis was generated using the PyMOL (v.2.5.4)-EasyMODELLER (v.4.0) software package. In all subpanels of **b**, the remaining icons were original creations or source icons created with Biorender.com. **c**, Gating for all single lymphocytes was based on granularity (FSC) and size (SSC). **d**, Staining of mononuclear cells with fluorescently-labeled antibodies: IgD-FITC; IgM-PE; IgA-APC; and CD19-PE-Cy7. The figures above show conventional bivariate scatterplots of side scatter signal versus cell marker-specific antibodies, with each plot derived from the preceding gate. In Supp. figs. **2c** and **2d,** the data acquisition and analyses were performed on BD FACSMelody™ cell sorter, while individual plots were produced using the BD FACSChorus™ software (v. 1.1.18.0).

a

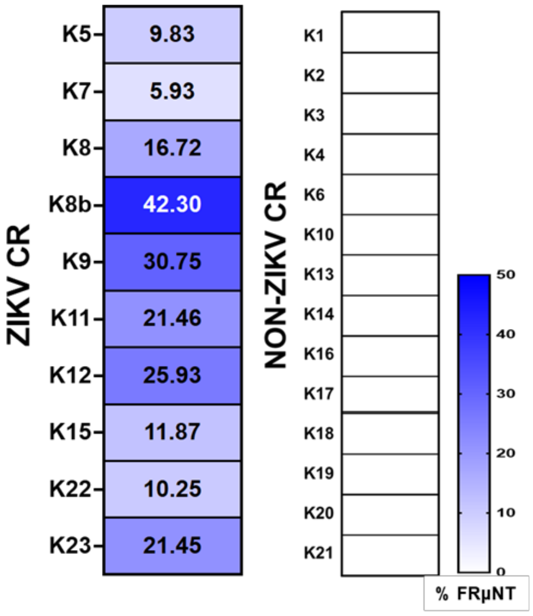

b

Comparison of binding ELISA endpoint titers relative to Isotype control						
huIgG ID	DENV-1	DENV-2	DENV-3	DENV-4	ZIKV	JEV
K5	*	***	***	***	**	***
K7	***	***	***	***	***	***
K8	***	***	***	***	***	***
K8b	***	***	***	***	***	***
K9	***	***	***	***	***	***
K11	***	***	***	***	***	***
K12	***	***	***	***	***	***
K15	***	***	***	***	***	***
K22	***	***	***	***	***	***
K23	***	***	***	***	***	***

c

Comparison of IC50 neutralization titers relative to Isotype control						
huIgG ID	DENV-1	DENV-2	DENV-3	DENV-4	ZIKV	JEV
K5	***	***	***	**	***	***
K7	*	***	ns	ns	ns	***
K8	ns	***	***	ns	ns	**
K8b	***	***	**	***	***	***
K9	**	***	**	ns	ns	***
K11	***	***	**	*	ns	***
K12	***	***	***	**	***	***
K15	***	***	***	*	ns	***
K22	ns	***	**	**	**	***
K23	ns	***	ns	ns	***	***

Supplementary Fig. 3: Screening of recombinantly-expressed human monoclonal antibodies from donor KH1891.

a, Neutralization screening against ZIKV PRVABC59 of 24 human monoclonal antibodies recovered from KH1891 from small-scale expression in mammalian cells. Non- and Neutralizing activity against ZIKV (expressed as %FRμNT) are shaded in white and blue, respectively. **b, c**, Statistical comparison of endpoint titers **(b)** and **(c)** IC50 neutralization titers of 10 purified huMAbs against DENV 1-4, JEV, and ZIKV VLPs and live, authentic viruses, respectively. Significant differences relative to the isotype (IgG1) control from three independent experiments were tested using one-way ANOVA followed by Bonferroni post-hoc tests. *, P<0.05; **, P<0.005; ***, P<0.0005.

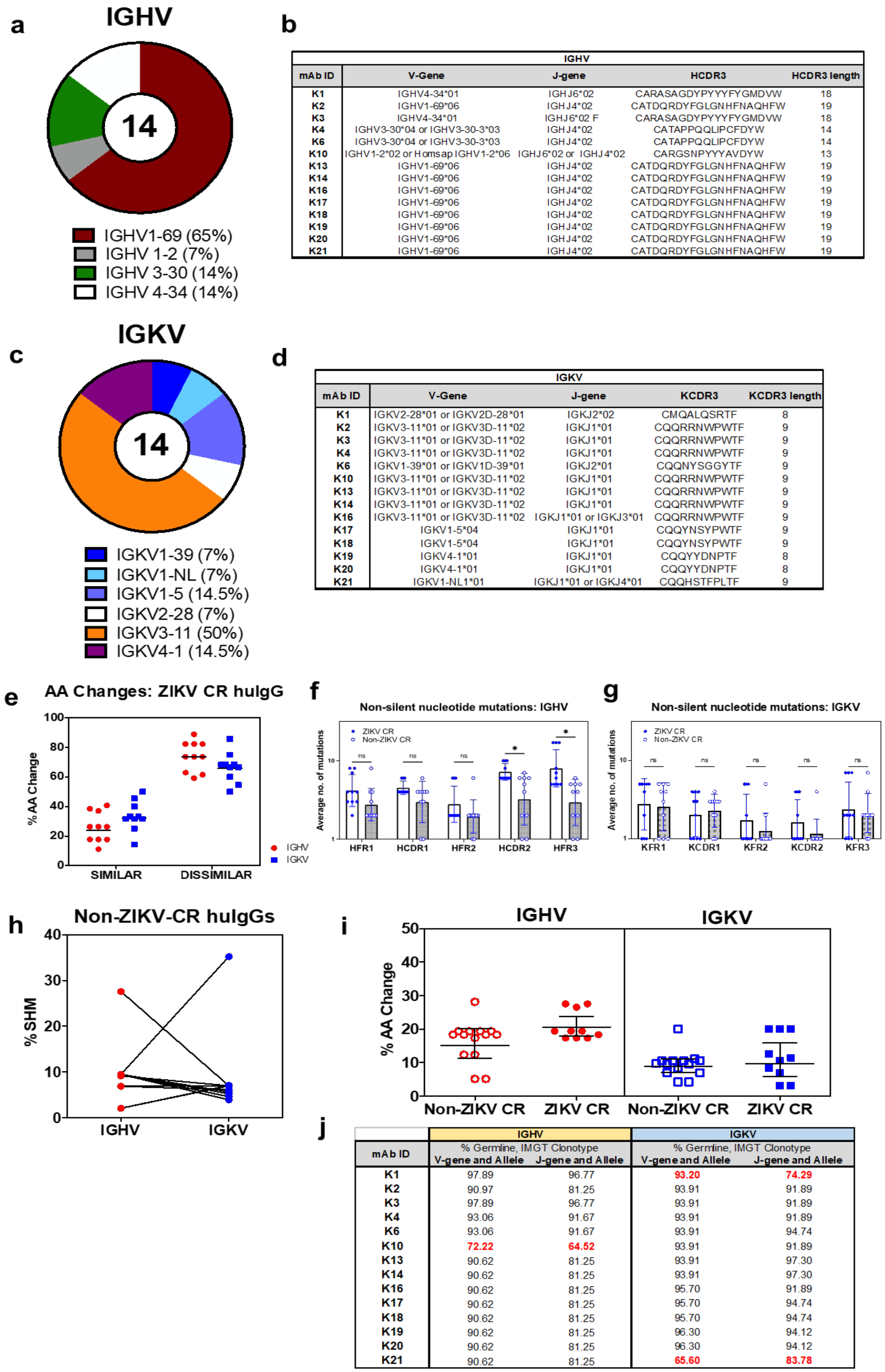

IGHV				
mAb ID	V-Gene	J-gene	HCDR3	HCDR3 length
K1	IGHV4-34*01	IGHJ6*02	CARASAGDYPYYYFYGMDVW	18
K2	IGHV1-69*06	IGHJ4*02	CATDQRDYFGLGNHFNAQHFW	19
K3	IGHV4-34*01	IGHJ6*02 F	CARASAGDYPYYYFYGMDVW	18
K4	IGHV3-30*04 or IGHV3-30-3*03	IGHJ4*02	CATAPPQQLIPCFDYW	14
K6	IGHV3-30*04 or IGHV3-30-3*03	IGHJ4*02	CATAPPQQLIPCFDYW	14
K10	IGHV1-2*02 or Homsap IGHV1-2*06	IGHJ6*02 or IGHJ4*02	CARGSNPYYYAVDYW	13
K13	IGHV1-69*06	IGHJ4*02	CATDQRDYFGLGNHFNAQHFW	19
K14	IGHV1-69*06	IGHJ4*02	CATDQRDYFGLGNHFNAQHFW	19
K16	IGHV1-69*06	IGHJ4*02	CATDQRDYFGLGNHFNAQHFW	19
K17	IGHV1-69*06	IGHJ4*02	CATDQRDYFGLGNHFNAQHFW	19
K18	IGHV1-69*06	IGHJ4*02	CATDQRDYFGLGNHFNAQHFW	19
K19	IGHV1-69*06	IGHJ4*02	CATDQRDYFGLGNHFNAQHFW	19
K20	IGHV1-69*06	IGHJ4*02	CATDQRDYFGLGNHFNAQHFW	19
K21	IGHV1-69*06	IGHJ4*02	CATDQRDYFGLGNHFNAQHFW	19

IGKV				
mAb ID	V-Gene	J-gene	KCDR3	KCDR3 length
K1	IGKV2-28*01 or IGKV2D-28*01	IGKJ2*02	CMQALQSRTF	8
K2	IGKV3-11*01 or IGKV3D-11*02	IGKJ1*01	CQQRRNWPWTF	9
K3	IGKV3-11*01 or IGKV3D-11*02	IGKJ1*01	CQQRRNWPWTF	9
K4	IGKV3-11*01 or IGKV3D-11*02	IGKJ1*01	CQQRRNWPWTF	9
K6	IGKV1-39*01 or IGKV1D-39*01	IGKJ2*01	CQQNYSGGYTF	9
K10	IGKV3-11*01 or IGKV3D-11*02	IGKJ1*01	CQQRRNWPWTF	9
K13	IGKV3-11*01 or IGKV3D-11*02	IGKJ1*01	CQQRRNWPWTF	9
K14	IGKV3-11*01 or IGKV3D-11*02	IGKJ1*01	CQQRRNWPWTF	9
K16	IGKV3-11*01 or IGKV3D-11*02	IGKJ1*01 or IGKJ3*01	CQQRRNWPWTF	9
K17	IGKV1-5*04	IGKJ1*01	CQQYNSYPWTF	9
K18	IGKV1-5*04	IGKJ1*01	CQQYNSYPWTF	9
K19	IGKV4-1*01	IGKJ1*01	CQQYYDNPTF	8
K20	IGKV4-1*01	IGKJ1*01	CQQYYDNPTF	8
K21	IGKV1-NL1*01	IGKJ1*01 or IGKJ4*01	CQQHSTFPLTF	9

	IGHV		IGKV	
	% Germline, IMGT Clonotype		% Germline, IMGT Clonotype	
mAb ID	V-gene and Allele	J-gene and Allele	V-gene and Allele	J-gene and Allele
K1	97.89	96.77	93.20	74.29
K2	90.97	81.25	93.91	91.89
K3	97.89	96.77	93.91	91.89
K4	93.06	91.67	93.91	91.89
K6	93.06	91.67	93.91	94.74
K10	72.22	64.52	93.91	91.89
K13	90.62	81.25	93.91	97.30
K14	90.62	81.25	93.91	97.30
K16	90.62	81.25	95.70	91.89
K17	90.62	81.25	95.70	94.74
K18	90.62	81.25	95.70	94.74
K19	90.62	81.25	96.30	94.12
K20	90.62	81.25	96.30	94.12
K21	90.62	81.25	65.60	83.78

Supplementary Fig. 4: Human Ig gene utilization and SHM analysis in non-ZIKV-CR human monoclonal antibodies.

a, c, Pie plots showing immunoglobulin heavy (a) and kappa light chain (c) gene family utilization and the distribution of fourteen DENV- or JEV-neutralizing human monoclonal antibodies (huMAbs) isolated from KH1891. The relative proportion of IGHV and IGκV gene usage is reported beside the different color schemes. **b, d**, Gene family assignments for the 11 non-ZIKV-CR huMAbs from KH1891. IMGT was used to assign the germline reference sequence for IGHV (b) and IGκV (d) and the relative similarity with the germline clonotype expressed in %. **e**, Proportion of non-silent mutations, which yielded amino acids with similar and dissimilar properties relative to the germline gene references among the ZIKV-CR huMAbs. **f, g**, Average number of non-silent nucleotide mutations distributed in the variable heavy (IGHV, **f**) and kappa light chain (IGκV, **g**) regions of ZIKV-CR and non-ZIKV-CR huMAbs. For **(f)** and **(g)**, statistical differences were tested using two-way ANOVA followed by Bonferroni post-hoc tests by comparing the average number of mutations in each group (n=10). Significant differences were denoted by an asterisk (*, $P<0.05$) or ns. CDR1-2, complementarity determining regions 1-2; FR1-4, framework region 1-4. **h**, Percentage (%) amino acid mutations in the IGHV and IGκV genes in DENV- or JEV huMAbs and ZIKV huMAbs. **i**, Comparison of the relative % amino acid (AA) changes in the paired IGHV and IGκV genes between ZIKV-CR huMAbs (n=10) and non-ZIKV-CR huMAbs (n=14). The black solid horizontal bars represent the geometric mean %AA changes with SD, analyzed within each group. No statistical significance was observed in all groups analyzed using one-way ANOVA followed by Tukey's multiple comparisons test, as defined by $P> 0.05$. **j**, Comparison of the V-J genes and alleles of heavy and light chains of non-ZIKV-CR huMAbs. Values highlighted in red show increased SHM (>20%) relative to the germline gene family.

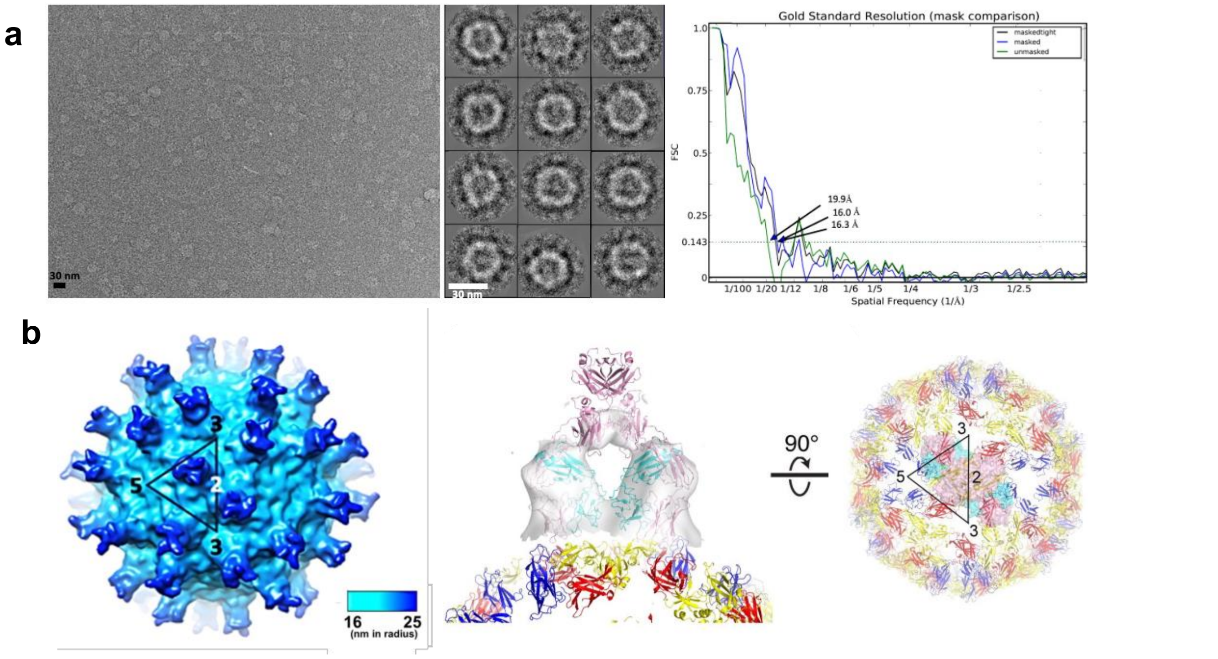

Supplementary Fig. 5: Cryo-EM data analysis and validation for K8b-IgG1 and mD2VLP complex.

a, Representative electron micrograph (**a, left**) and 2D-class averages (**a, middle**) of the mD2VLP-K8b-IgG1 immune complex with a scale bar of 30 nm. The gold-standard Fourier shell correlation curve of the final refined maps for the K8b-IgG1 and mD2VLP complex and the resolution estimation based on the 0.143 Fourier shell correlation criteria (**a, right**). **b**, The appearance of Fc in the cryo-EM structure of K8b-IgG1 and mD2VLP complex rendered at a threshold of 1.2 σ (**b, left**) was a guide for determining the antibody orientation and pairing. The two Fab regions, representing a 2-fold symmetry, were identified as a pair. The side view (**b, middle**) and top view (**b, right**) of the complex illustrated the potential binding mode of the K8b-IgG1-mD2VLP complex. The full-length K8b was shown as ribbon models (chain H in pink and chain L in cyan). The EM density around the K8b-IgG1 structure was represented as a gray surface. The full-length K8b-IgG1 was shown as a transparent surface model. One icosahedral asymmetric unit was outlined as a triangle.

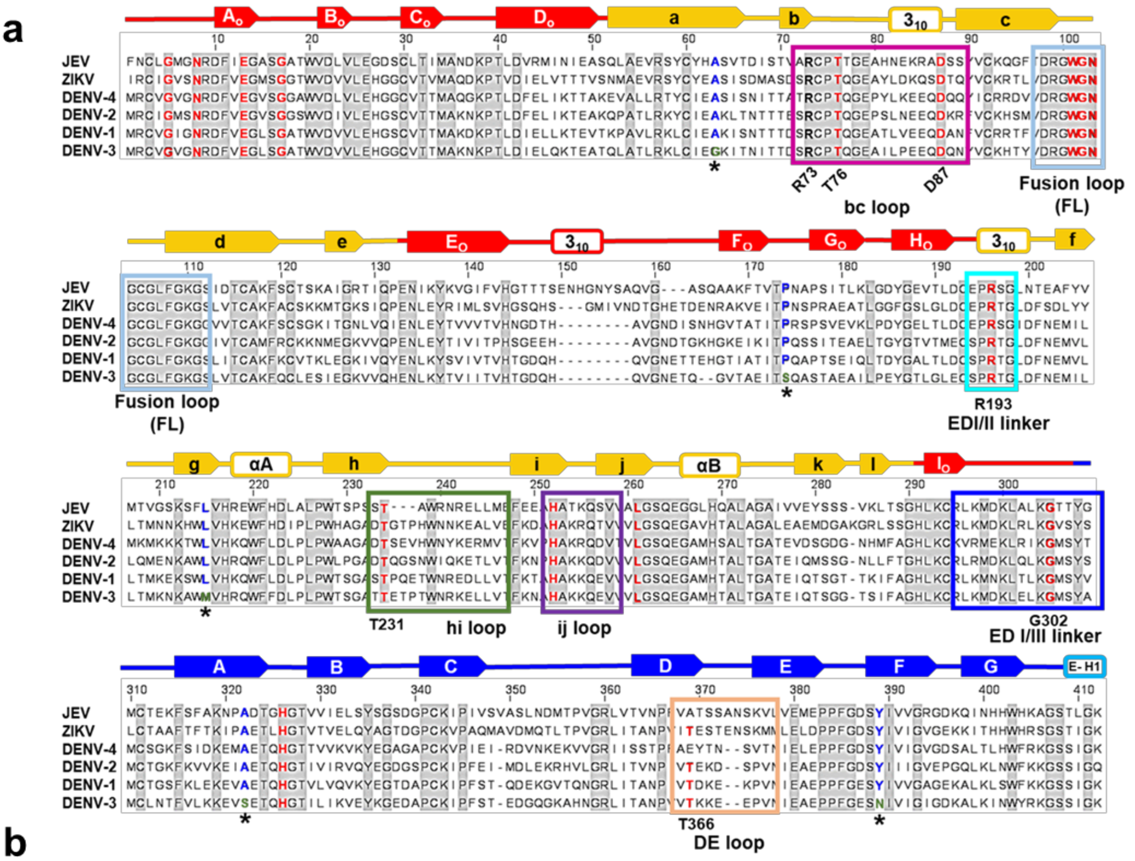

b

AA	Residue No.	AA Residue	SASA	E domain	location
ARG	73	R73	0.0668	DII	bc loop
THR	76	T76	0.650	DII	bc loop
ASP	87	D87	0.360	DII	bc loop
ARG	193	R193	0.632	DI/II hinge	ED I/II linker
THR	231	T231	0.995	DII	hi loop
GLY	302	G302	0.0559	D I/III hinge	loA loop
THR	366	T366	0.697	DIII	DE loop

			SASA values#					
E Region	Amino acid (AA)	Site §	DENV-1	DENV-2	DENV-3	DENV-4	JEV	ZIKV
b-c loop	ARG (R)	**R73**	0.011	0.087	0.069	0.031	0.037	0.067
	THR (T)	**T76**	0.010	**0.34**	0.088	0.081	0.15	**0.65**
	ASP (D)	**D87**	**0.38**	**0.52**	0.027	**0.45**	0.26	**0.36**
ED I/II linker	ARG (R)	**R193**	**0.51**	**0.52**	**0.78**	**0.49**	**0.57**	**0.63**
hi loop	THR (T)	**T231**	**0.75**	**0.68**	**0.58**	**0.57**	**0.75**	**1.00**
loA loop	GLY (G)	**G302**	0.011	0.16	0.019	0.011	0.024	0.056
DE loop	THR (T)	**T366**	**0.31**	**0.34**	**0.63**	**0.55**	**0.44**	**0.70**

§ AA residue numbering according to the ZIKV
# Solvent-accessible surface area (SASA) values highlighted in bold and in red are surface-exposed (QSASA >0.30)

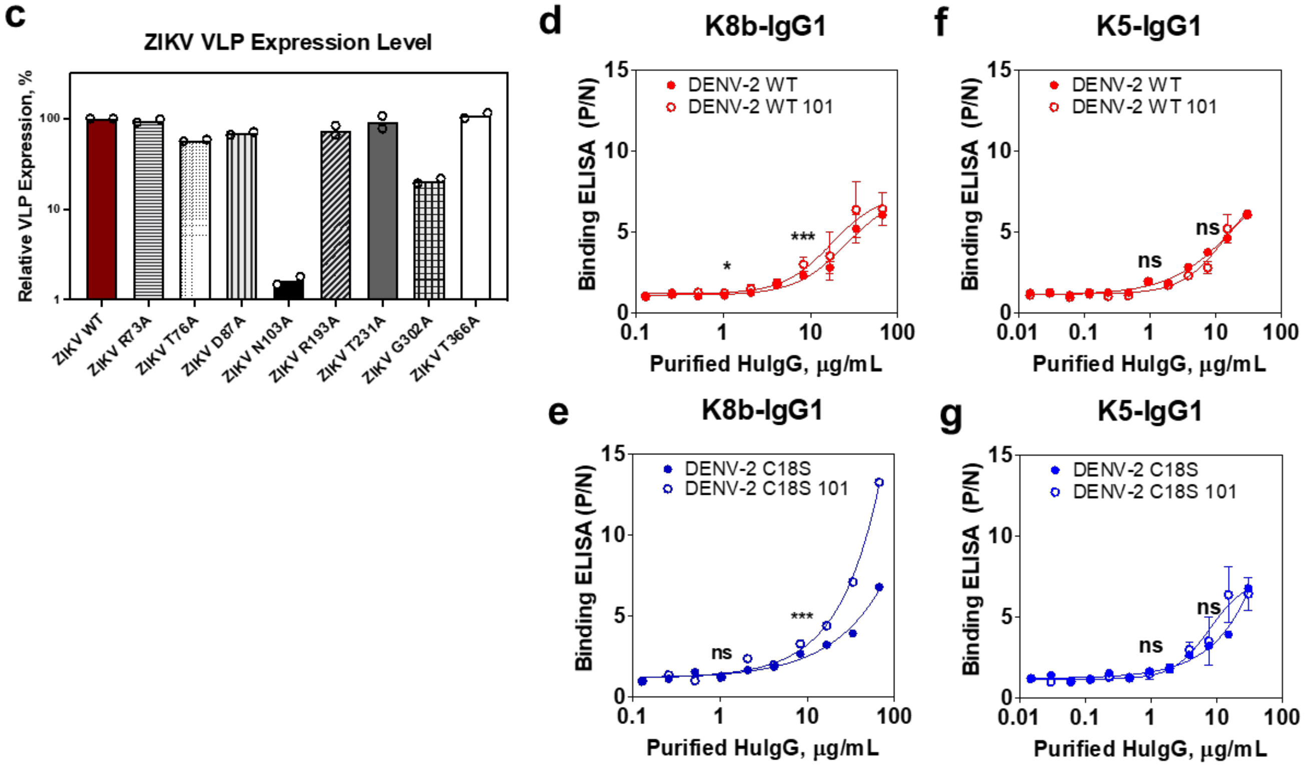

Supplementary Fig. 6: Epitope mapping of K8b and K5.

a, Amino acid sequence alignment of the E proteins among prototype flaviviruses including selected DENV serotypes, Japanese encephalitis virus (JEV), and Zika virus (ZIKV) using Clustal Omega and visualized by JalView 2.11.1.4. Sequence identities were normalized by aligned length, while the positions of the amino acids in E are labeled according to ZIKV PRVABC59. The shaded residues show 100% conservancy among the selected flavivirus genera. Above the sequences, the arrows indicate β-strands, rectangles represent the helices, and lines show the spanning loops and strands in the E structure. The E domains I, II, and III are represented by arrows and lines in red, yellow, and blue, respectively. Potential binding sites of K8b are highlighted in boxes: bc loop (magenta), fusion loop (FL, light blue), EDI/II linker region (cyan), hi loop (green), ij loop (purple), I_oA loop or the EDI/III linker region (blue), and DE loop of EDIII (flesh). **b**, Tabulated list of surface-exposed AA residues and consensus among DENV serotypes 1-4, JEV, and ZIKV, including their E domain and specific location. The seven (7) amino acid residues that have been mutated to alanine **(b, left)** and their corresponding surface-accessible surface area (SASA) values in DENV-1, DENV-2, DENV-3, DENV-4, JEV, and ZIKV **(b, right)**. In Supp. Figs. **6a** and **6b**, residues in bold and in red are surface-exposed (QSASA>0.30). **c**, Expression levels of mutant ZIKV VLPs relative to the ZIKV wild-type (WT), expressed as %. The vertical axis shows the relative expression levels of each ZIKV VLP mutant as the geometric mean (n=2) relative to the ZIKV WT VLP. Bars represent the geometric means of two replicates, represented by empty spheres. **d-g**, Binding ELISA profile of purified **K8b (d, e)** and **K5 (f, g)** against DENV-2 WT (d, f) and mD2VLP (e, g) and their corresponding mutants, mutated at W101. WT and mutant VLPs are shown in shaded and empty circles, respectively. The significant difference in binding activities of K8b to each pair of VLPs is shown with asterisks and was taken from two independent experiments. For K8b **(d)**, the p values at 1.0 µg/mL (*, $P<0.05$) and 10 µg/mL (***, $P<0.001$) were determined on the geometric mean of log-transformed data using one-way ANOVA with Tukey's multiple comparisons post-test, and were found to be significant relative to the wild-type VLPs; the level of significance is similarly defined for **(e)** at ***, $P<0.001$. For **K5 (f, g)**, the p values at 1.0 µg/mL and 10 µg/mL were determined on log-transformed data using one-way ANOVA with Tukey's multiple comparisons post-test and showed no significant difference in binding relative to the wild-type VLPs (ns, $P>0.05$).

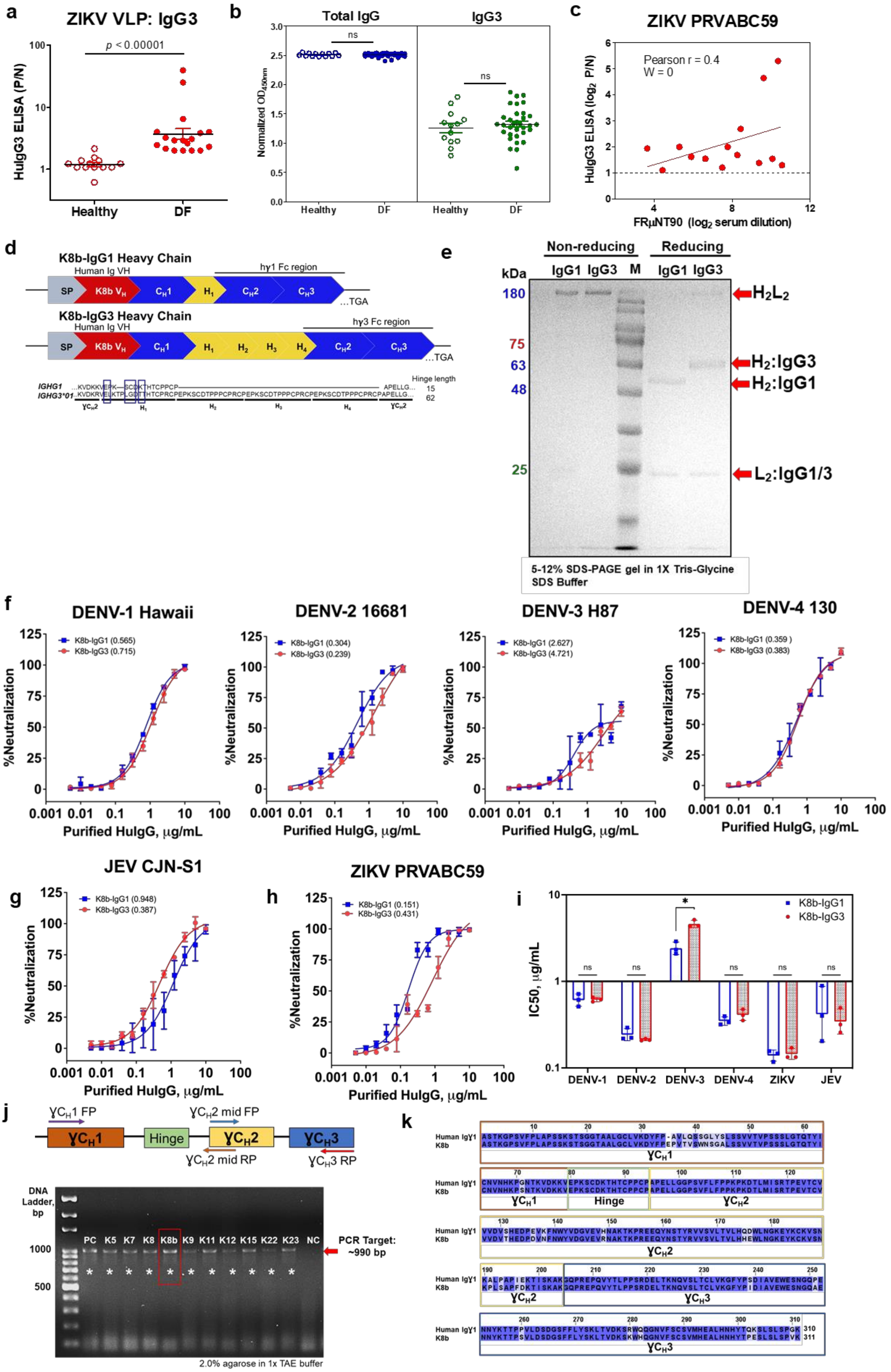

Supplementary Fig. 7: Human IgG3-focused responses in JEV-immune, dengue-infected individuals and Neutralization profile of K8b expressed as IgG1 and IgG3.

a, ZIKV VLP-reactive human IgG3 antibodies from healthy (n=13) and DF (n=18) donors. DF individuals are shown to have elevated human IgG3 antibodies against ZIKV VLP with significantly higher geometric mean titer compared to healthy donors where $P<0.00001$. The average individual titers of healthy and DF donors are shown in empty and red-filled circles, respectively. **b**, Detection of total human IgGs and relative IgG3-specific antibodies among healthy (n=13) and dengue-infected febrile (DF, n=33) individuals. Normalized ELISA OD values of healthy donors are shown in empty blue or green circles, and those of DF individuals are in blue- or green-filled dots. No significant differences were shown in geometric mean ELISA binding titers for each group (ns, $P>0.05$). **c**, Correlation between the ZIKV VLP-binding IgG3 subclass and ZIKV neutralization titers of DF individuals, with a significant difference, indicated as $P<0.05$ by Pearson's correlation (Pearson's r=0.4; Wilcoxon matched-pairs signed rank test, W=0; $P<0.05$). The ZIKV VLP was properly titrated to obtain equimolar concentrations per well before use in all antigen-capture ELISAs. Black solid horizontal bars represent the geometric mean titers (GMT) ± standard deviations (SD) of samples in binding ELISA expressed as normalized OD or P/N values analyzed within each group. All data are representative of three independent experiments. ZIKV=Zika virus; VLP=virus-like particle; FRμNT=focus reduction microneutralization test. **d**, Schematic representation of the designed gene constructs of K8b expressed as full-length human IgG1 (K8b-IgG1) and IgG3 (K8b-IgG3). Below the construct is the pairwise comparison of the amino acid (aa) sequences of the hinge and selected CH2-CH3 regions of K8b IgG1 and IgG3 using Clustal Omega, highlighting the differences in length (IgG1=15aa; IgG3=62aa) and composition of the hinge region (IgG1=1; IgG3=4). Highlighted in blue boxes are the differing AA residues between the two isotypes. **e**, SDS-PAGE of the purified human monoclonal antibody, K8b, expressed as full-length IgG1 or full-length IgG3 visualized under reducing and non-reducing conditions. The purity of the preparations and the expected 10 kDa higher molecular weight of K8b-IgG3 than K8b-IgG1 is highlighted by the red arrow marks. **f-h**, *In vitro* ZIKV microneutralization assay profiles of purified K8b expressed as IgG1 and IgG3 against the prototype strains of DENV-1 to -4 **(f)**, JEV **(g)**, and ZIKV **(h)**. Each point in the curve shows the mean ± SD of data from three independent experiments. The geometric mean half-maximal inhibitory concentration (IC50) for each isotype is expressed at μg/mL and indicated in parenthesis. **i**, Comparison of the geometric mean ± SD IC50 titers between K8b-IgG1 and K8b-IgG3 against the prototype virion strains tested from three independent experiments. Significant differences for each data pair were determined on the geometric mean of log-transformed data using two-way ANOVA with Šídák's multiple comparisons post-test, and was found to be significant for DENV-3 IC50 titers represented by an asterisk (*, $P<0.05$). No significant differences were observed in other groups, as indicated by ns ($P>0.05$). Individual data points for K8b-IgG1 (blue) and K8b-IgG3 (red) are also shown in colored squares and spheres, respectively. **j**, Schematic representation of the strategy to amplify the human Fc-region or Immunoglobulin (Ig) gamma (Ɣ) constant region including domains 1-3 (ƔC_H1-3) and the hinge. Oligonucleotide primers targeting the Ig-gene-specific regions are also shown and are represented by arrows. The agarose gel electrophoresis (AGE) image below shows the PCR amplicons from the human IgƔ region of the ten ZIKV-CR B cells of donor KH1891. Each well contains the PCR products of the ZIKV-CR IgƔ genes except for the far left lane that contains the 100bp-DNA ladder, the positive control (PC, lane 2), and the PCR negative sample control (NC, far right lane). Asterisk (*) indicates a positive PCR result. **k**, Pairwise comparison of amino acids of K8b with the closest matching human Ig gamma-1 chain C region (human IgƔ1, NCBI Sequence ID: AK125633.1) using Clustal Omega and visualized by JalView 2.11.1.4. IgBLAST analysis showed 283 out of 311 (91%) aa identity. Sequence identities were normalized by aligned length, while the amino acid positions were labeled based on K8b. The residues shaded in dark blue show 100% conservancy between the two sequences, while those highlighted in light blue or white represent 50% and 0% identity, respectively. The human Ig gamma constant (ƔC_H) and hinge regions are highlighted in colored boxes: ƔCH1 (mahogany), hinge (green), ƔC_H2 (yellow), and ƔC_H3 (blue).

a

E domain		DII	DI	DII	DIII	DIII
	Conserved	A	P	L	A	Y
IMGT® Classification	Very Similar / Similar			M		
	Dissimilar	G	S		S	
	Very Dissimilar					N
AA residue position	DENV-1, -2, -4	63	166	207	313	377
	DENV-3	63	164	205	311	375
	JEV	63	171	212	315	382
	ZIKV	63	171	212	319	386

AA Residue	E domain	Location
A	DII	a strand
H	DI	ß strand: Fo loop
P	DI	ß strand: Fo loop
T	DI	G-H loop
L	DII	f-g loop
L	DII	g-αA loop
G	DIII	DI/DIII linker
A	DIII	A strand
E	DIII	A-B loop
Y	DIII	F strand
L	DIII	F strand
F	DIII	G strand

b

E domain		DII	DI	DI	DI	DII	DII	DIII	DIII	DIII	DIII	DIII	DIII
	Conserved	A	H	P	T	L	L	G	A	E	Y	L	F
IMGT® Classification	Very Similar											I	
	Similar					M							
	Dissimilar	G		S	G		F		S				Y
	Very Dissimilar							N		K	N		
AA residue position	DENV-1, -2, -4	63	158	166	182	207	214	304	313	327	377	389	392
	DENV-3	63		164	180	205	212	302	311	325	375	387	390

c

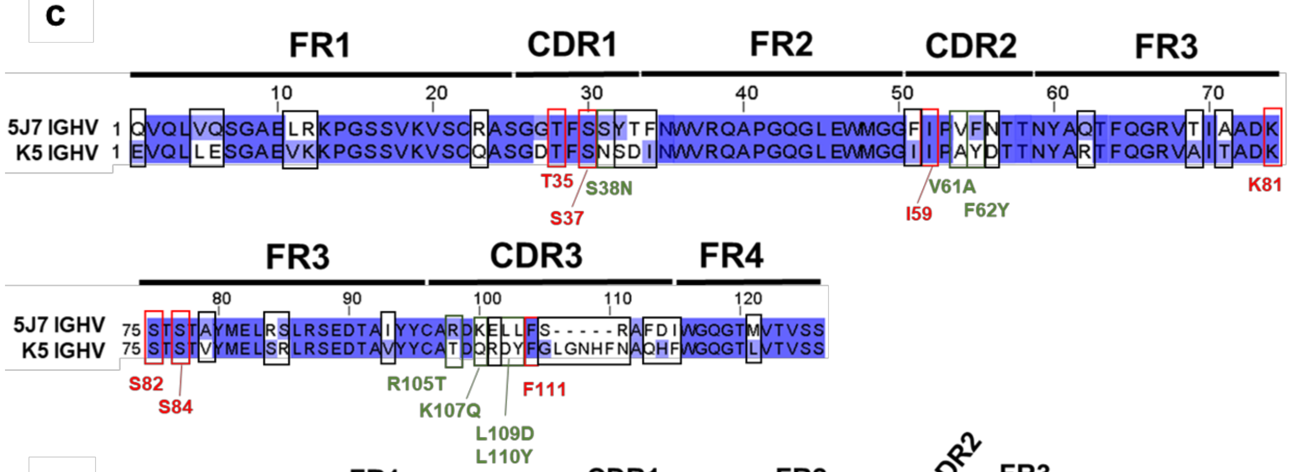

d

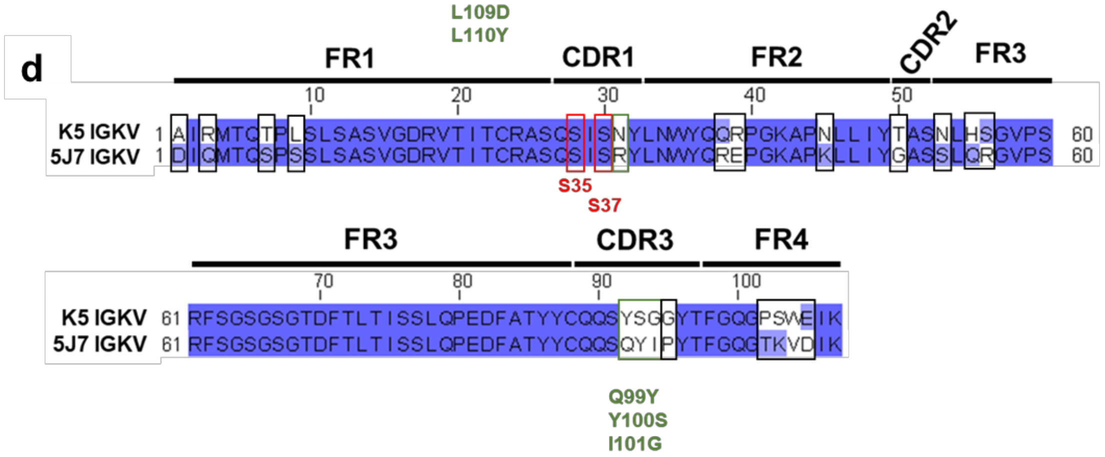

Supplementary Fig. 8: Flavivirus E sequence and huMAb K5 sequence analyses.

a, b, Comparison of the flavivirus sequences showing differential amino acid residues among the (**a**) DENV 1-4 serotypes, JEV, and ZIKV virions and (**b**) the within DENV serocomplex. Colored boxes represent the conservancy (white) of amino acids (AA), and the non-silent AA changes as they belong to similar (salmon) or dissimilar (green) classes based on IMGT® classifications. The corresponding AA positions on the virion are also shown below the AA grouping. **c, d**, Pairwise comparison of the amino acid (AA) sequences in the immunoglobulin heavy (IGHV) (**c**) and Kappa light (IGκV) (**d**) chain variable domains of huMAbs 5J7 and K5. The 5J7 AA residues reportedly interacting with the E protein, which were consensus or dissimilar with that of K5, are enclosed in red and green boxes and texts, respectively. Dissimilar AA residues between 5J7 and K5 are shown in black boxes. The AA numbering in both Ig variable antibody regions followed the 5J7 numbering as published (Fibriansah *et al.*, 2015). CDR1-3, complementarity determining regions 1-3; FR1-4, framework region 1-4.

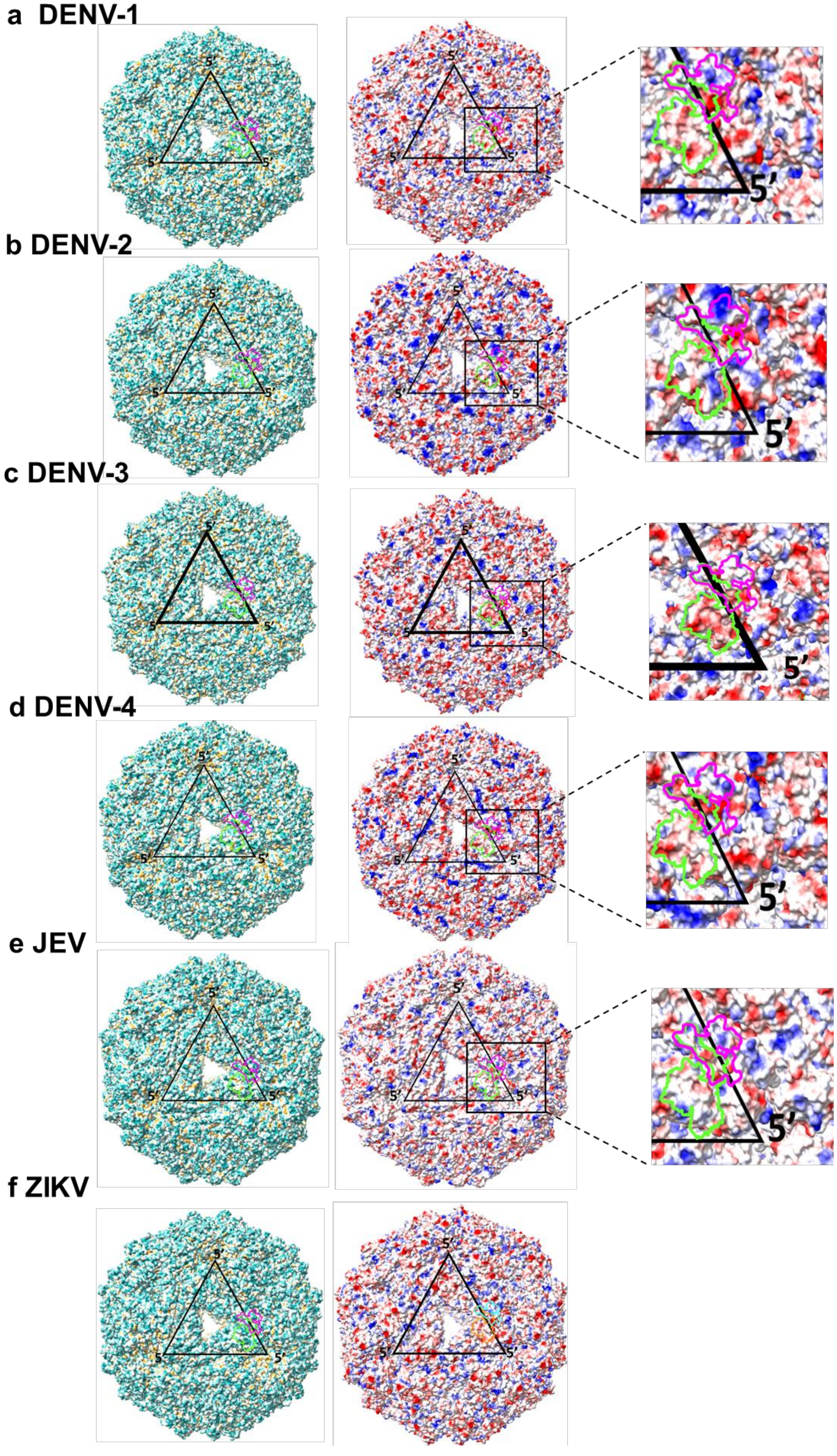

Supplementary Fig. 9: Electrostatic charges and Hydrophobicity of the four DENV serotypes, JEV, and ZIKV VLPs.

Comparison of the electrostatic charges and hydrophobic residues of the K8b epitope across various flavivirus VLP structures. The hydrophobicity (**left**) and electrostatic potential surface maps (**middle**) of (**a**) DENV-1, (**b**) DENV-2, (**c**) DENV-3, (**d**) DENV-4, (**e**) JEV, and (**f**) ZIKV. The contour levels are around 60 +/- kT/e. In all images, the landscape of the putative binding sites of K8b-IgG1 variable heavy and kappa light chain domains are mapped on all VLPs in green and pink contour lines, respectively. The black lines forming a triangle define an asymmetric unit in a ZIKV VLP structure at T=1. Icosahedral five-fold (5') symmetry elements are labeled. **(right)** A magnified view of the surface rendering of solvent-exposed residues in each VLP three-dimensional (3D) structure, where highly conserved and surface-exposed residues are colored red and buried residues are colored blue. The images were generated using the PyMOL (v.2.54)-MODELLER (v.4.0) software package.

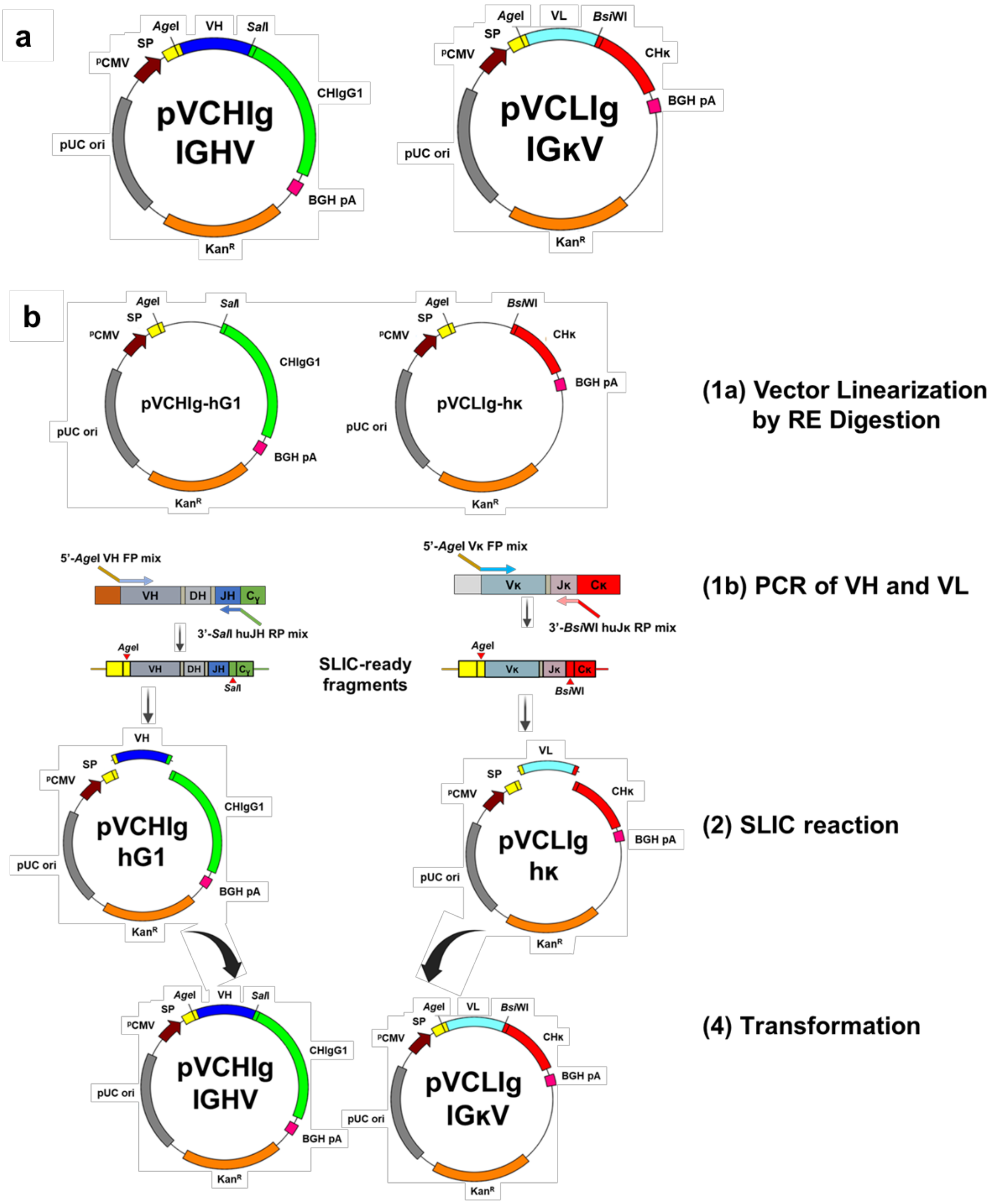

Supplementary Fig. 10: Generation of in-house expression vectors for antibody production.

a, Map of the mammalian expression plasmids, pVCHIg-hG1 and pVCLIg-hκ, for the recombinant expression of the heavy-chain and Kappa light chain genes; **b**, Diagram of the steps leading to the successful cloning of the human immunoglobulin (Ig) heavy and light chain variable (V) gene domains. Expression vectors were independently linearized using restriction enzymes, as shown above, prior to the homologous recombination of the human Ig V-genes using sequence and ligation-independent cloning (SLIC) as a cloning strategy. SLIC was followed by a standard transformation protocol. The plasmid maps in all panels in this figure were generated using an open-source plasmid visualization software, Plasm (v.2.0).

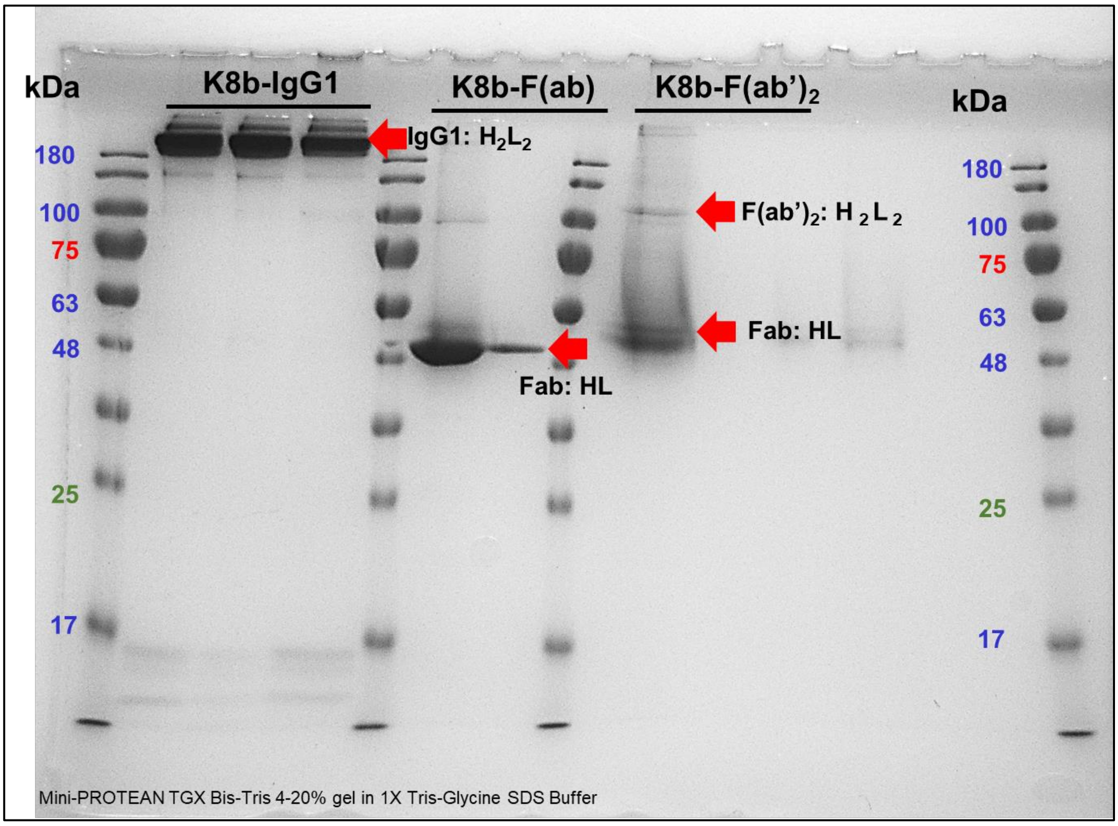

Supplementary Fig. 11: Uncropped SDS-PAGE Gel electrophoretic separation of the purified human monoclonal antibody, K8b, expressed as IgG1, F(ab), and F(ab)'$_2$ under non-reducing conditions, as originally presented in Fig. 5b.

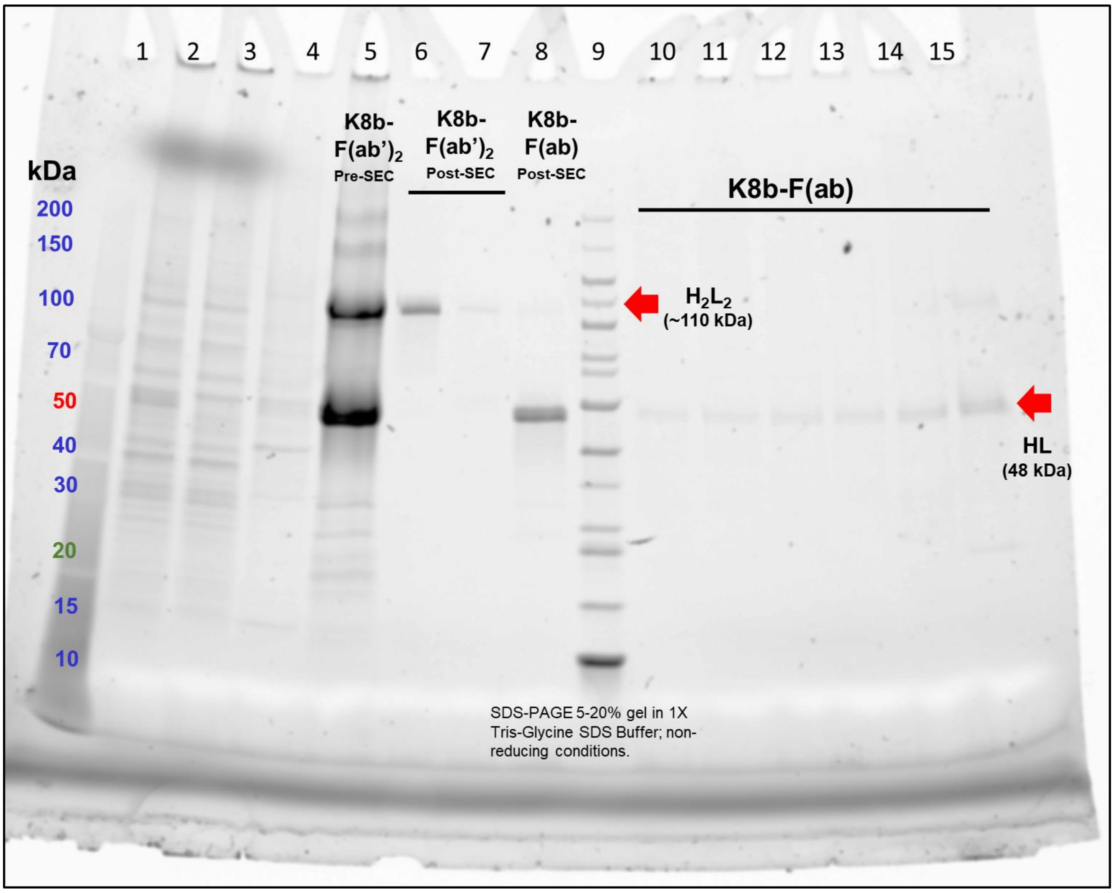

Supplementary Fig. 12: Uncropped SDS-PAGE gel separation of the purified K8b-F(ab)'$_2$ molecules and K8b-F(ab) molecules after size-exclusion chromatography, as originally presented in Fig. 5c. Other samples were included in the run, whose identities were identified with a legend in numbered wells. Lanes: 1, 9: MW marker; 2-4: unpurified cell culture supernatant containing K8b-F(ab')$_2$; 5: post-purification pooled fractions of K8b-F(ab')$_2$ pre-SEC; 6: K8b-F(ab')$_2$ post-SEC; 7: wash FT of K8b-F(ab')$_2$ (post-SEC); 8: K8b-F(ab) molecules post-SEC; 10-15: K8b-F(ab) fractions (post-SEC wash).

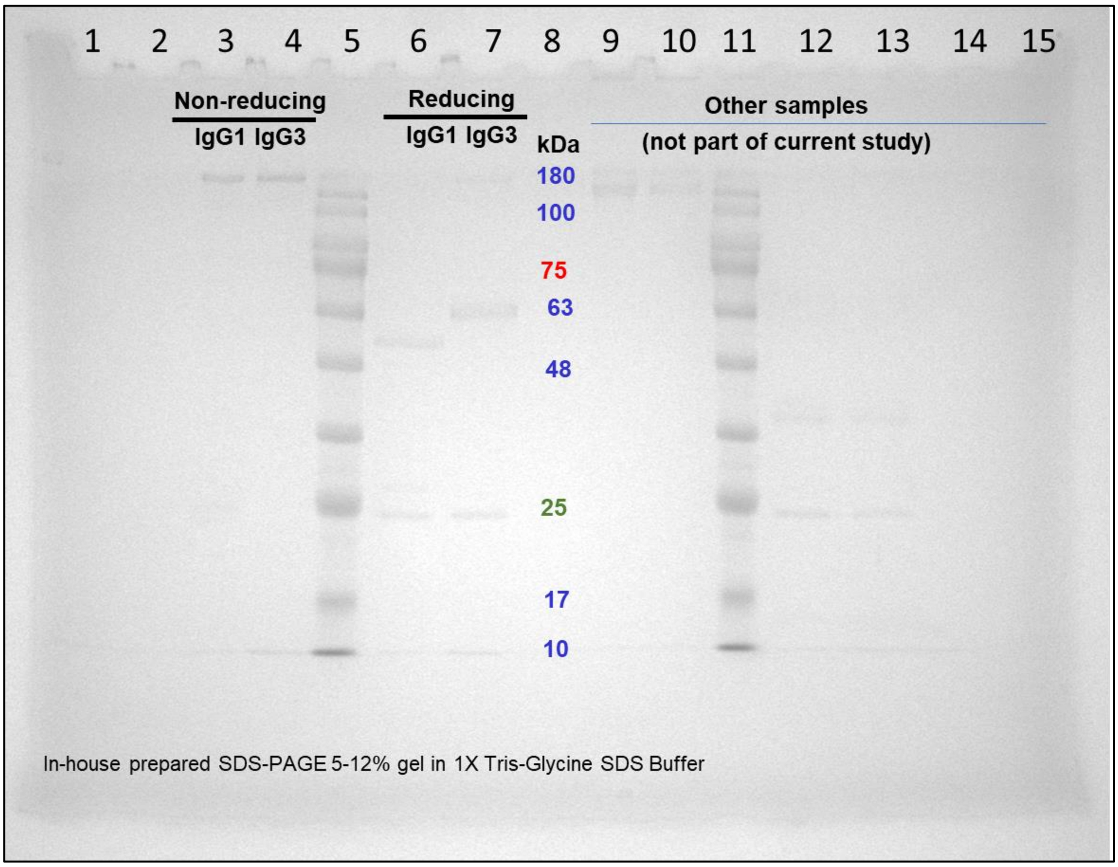

Supplementary Fig. 13: Uncropped SDS-PAGE of the purified human monoclonal antibody, K8b, expressed as full-length IgG1 or full-length IgG3 visualized under reducing and non-reducing conditions. as originally presented in Supp. Fig. 7e. Other samples were included in the run, whose identities were identified with a legend in numbered wells. Lanes: 1,2, and 8: Empty lanes; 3: K8b-IgG1 (non-reducing condition); 4: K8b-IgG3 (non-reducing condition); 5: MW marker; 6: K8b-IgG1 (reducing condition); 7: K8b-IgG3 (reducing condition); and 9~15: other samples (not part of the current study).

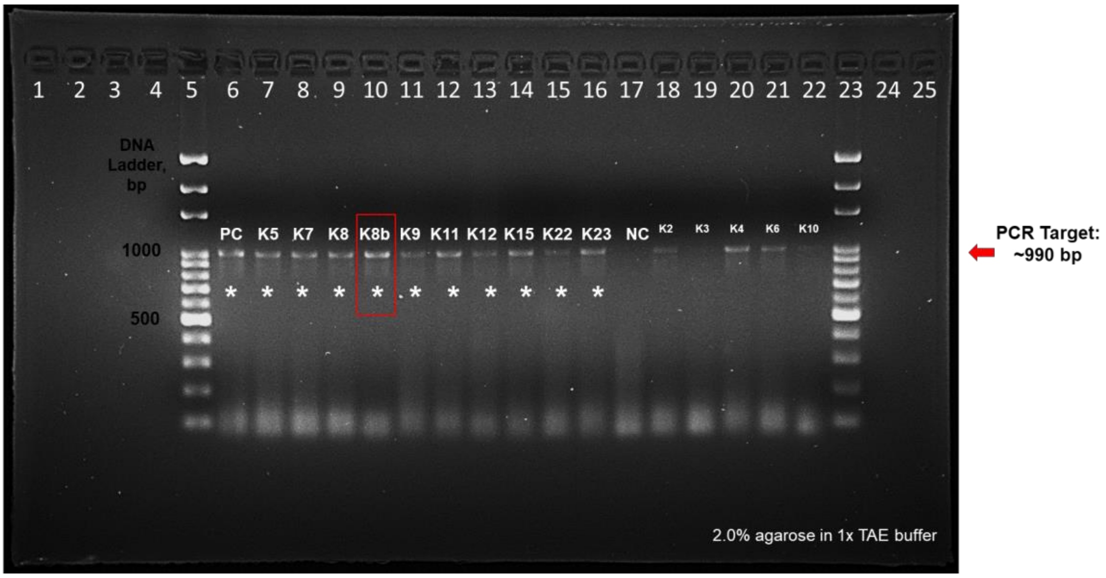

Supplementary Fig. 14: Uncropped agarose gel electrophoresis (AGE) image showing the PCR amplicons from the human Igɣ region of the ten ZIKV-CR B cells of donor KH1891, as originally presented in Supplementary Fig. 7j. Other samples were included in the run, whose identities were identified with a legend in numbered wells. Lanes: 1~4, 24, and 25: empty lanes; 5, 23: DNA ladder, 100 bp; 6: PCR positive control (PC); 7:K5; 8:K7; 9:K8; 10: K8b; 11:K9; 12: K11; 13:K12; 14:K15; 15:K22; 16:K23; 17: PCR Negative control (NC); and 18~22: other Non-ZIKV CR.
