# Supplementary material for: Antibodies from dengue patients with prior exposure to Japanese encephalitis virus are broadly neutralizing against Zika virus
Source: Commun Biol. 2024 Jan 24;7:15. doi: 10.1038/s42003-023-05661-w (PMC10808242; doi:10.1038/s42003-023-05661-w)
Supplement: Supplementary file 3 — Description of Additional Supplementary Files [file 42003_2023_5661_MOESM3_ESM.pdf]

Description of Additional Supplementary Files

File Name: Supplementary Data 1
Description: Panel of healthy and dengue-infected plasma at the acute phase with the corresponding DENV, JEV, YFV, and ZIKV neutralization profiles.

File Name: Supplementary Data 2
Description: Oligonucleotide primers used for the generation of various in-house antibody expression vectors.

File Name: Supplementary Data 3
Description: Human monoclonal antibody isolation primers used in this study.

File Name: Supplementary Data 4
Description: Universal primers used for the amplification of human Ig gamma constant domains.

File Name: Supplementary Data 5
Description: Primers used for site-directed mutagenesis on ZIKV VLP.

File Name: Supplementary Data 6
Description: Source data underlying main figures.
