# Supplementary material for: Antibodies from dengue patients with prior exposure to Japanese encephalitis virus are broadly neutralizing against Zika virus
Source: Commun Biol. 2024 Jan 24;7:15. doi: 10.1038/s42003-023-05661-w (PMC10808242; doi:10.1038/s42003-023-05661-w)
Supplement: Supplementary file 6 — Reporting Summary [file 42003_2023_5661_MOESM6_ESM.pdf]

nature portfolio

COMMSBIO-23-3724-T
Corresponding author(s): Day-Yu Chao

Last updated by author(s): 10/02/2023

Reporting Summary

Nature Portfolio wishes to improve the reproducibility of the work that we publish. This form provides structure for consistency and transparency in reporting. For further information on Nature Portfolio policies, see our Editorial Policies and the Editorial Policy Checklist.

Statistics

For all statistical analyses, confirm that the following items are present in the figure legend, table legend, main text, or Methods section.

n/a	Confirmed	
☐	☒	The exact sample size (*n*) for each experimental group/condition, given as a discrete number and unit of measurement
☐	☒	A statement on whether measurements were taken from distinct samples or whether the same sample was measured repeatedly
☐	☒	The statistical test(s) used AND whether they are one- or two-sided<br>*Only common tests should be described solely by name; describe more complex techniques in the Methods section.*
☒	☐	A description of all covariates tested
☒	☐	A description of any assumptions or corrections, such as tests of normality and adjustment for multiple comparisons
☐	☒	A full description of the statistical parameters including central tendency (e.g. means) or other basic estimates (e.g. regression coefficient) AND variation (e.g. standard deviation) or associated estimates of uncertainty (e.g. confidence intervals)
☐	☒	For null hypothesis testing, the test statistic (e.g. *F*, *t*, *r*) with confidence intervals, effect sizes, degrees of freedom and *P* value noted<br>*Give P values as exact values whenever suitable.*
☒	☐	For Bayesian analysis, information on the choice of priors and Markov chain Monte Carlo settings
☒	☐	For hierarchical and complex designs, identification of the appropriate level for tests and full reporting of outcomes
☒	☐	Estimates of effect sizes (e.g. Cohen's *d*, Pearson's *r*), indicating how they were calculated

Our web collection on statistics for biologists contains articles on many of the points above.

Software and code

Policy information about availability of computer code

Data collection	Data points were collected based on each experimental results with the minimum of two repeats.<br>Flow cytometry data: Operating system software for BD FACSMelody™ Cell sorter (Becton, Dickinson, and Company)<br>CryoEM image processing: EMAN2
Data analysis	Graph plotting and statistical analysis: GraphPad Prism (version 9.5.1, GraphPad Software Inc.)<br>FACS Analysis: BD FACSChorus™ software<br>DNA sequence visualization: SnapGene v6.2.2<br>Immunoassays: XFluor4 v4.51 software (Tecan Sunrise)<br>Immunecomplex 3D reconstruction and structure prediction: EMAN2 and UCSF ChimeraX (version 1.3)<br>Antibody epitope prediction: MODELLER (v10.4)<br>Antigen-antibody interfaces: PDBePISA30 and PDBsum (online service provided by EMBL-EBI)<br>E protein amino acid sequence alignment: EasyModeller v2.2.6 and JalView (v2.11.1.4)<br>Solvent accessibility calculation: POPS<br>Virus-like particles structure prediction of five different flaviviruses (JEV, ZIKV, DENV-1, DENV-3, DENV-4) based on mD2VLP (EMD-6926): PyMOL using in-house scripts, which is available upon request

For manuscripts utilizing custom algorithms or software that are central to the research but not yet described in published literature, software must be made available to editors and reviewers. We strongly encourage code deposition in a community repository (e.g. GitHub). See the Nature Portfolio guidelines for submitting code & software for further information.

Data

Policy information about availability of data

All manuscripts must include a data availability statement. This statement should provide the following information, where applicable:

- Accession codes, unique identifiers, or web links for publicly available datasets
- A description of any restrictions on data availability
- For clinical datasets or third party data, please ensure that the statement adheres to our policy

All data that support the findings of this study are available in the supplementary data or upon reasonable request.

Research involving human participants, their data, or biological material

Policy information about studies with human participants or human data. See also policy information about sex, gender (identity/presentation), and sexual orientation and race, ethnicity and racism.

Reporting on sex and gender	No sex and gender-based analysis were performed in this study.
Reporting on race, ethnicity, or other socially relevant groupings	No race, ethnicity or other socially relevant groupings were identified and analyzed in this study.
Population characteristics	We only considered age differences among the study participants to identify which population was covered by the national pediatric vaccination strategy against Japanese encephalitis virus implemented in Taiwan starting 1968.
Recruitment	All subjects were recruited in 2014-2015, during the two largest DENV-1 and DENV-2 outbreaks in southern Taiwan. Subjects with febrile symptoms suspected of dengue viral infection during the acute phase (i.e., two weeks post-infection (poi)) were enrolled in an on-going study of the Kaohsiung Medical University.
Ethics oversight	Institutional Review Board (IRB) of Kaohsiung Medical University (IRB No. KMUHIRB-E (II)-20180092)

Note that full information on the approval of the study protocol must also be provided in the manuscript.

Field-specific reporting

Please select the one below that is the best fit for your research. If you are not sure, read the appropriate sections before making your selection.

☒ Life sciences ☐ Behavioural & social sciences ☐ Ecological, evolutionary & environmental sciences

For a reference copy of the document with all sections, see nature.com/documents/nr-reporting-summary-flat.pdf

Life sciences study design

All studies must disclose on these points even when the disclosure is negative.

Sample size	The human plasma samples comprised 60 dengue-confirmed individuals and 80 dengue-negative controls. The sample sizes were determined based on the availability of the archived samples and a minimum of 30 subjects per age group for normal distribution.
Data exclusions	No exclusion criteria were pre-established, and no collected data were excluded from the analysis.
Replication	Data presented were replicated in at least three independent experiments with similar results.
Randomization	For the human plasma samples, no randomization was pre-established since this is an observational study and all samples were allocated to either dengue or non-dengue group depending on the confirmatory laboratory diagnostic results. For in vivo study, the allocation of mice to experimental groups was random.
Blinding	No blinding of the human subjects was pre-established in this study since this is an observational study. However, for practical reasons, investigators were not blinded to the prime-boost immunization regimen administered to mice in the experiments.

Reporting for specific materials, systems and methods

We require information from authors about some types of materials, experimental systems and methods used in many studies. Here, indicate whether each material, system or method listed is relevant to your study. If you are not sure if a list item applies to your research, read the appropriate section before selecting a response.

Materials & experimental systems

n/a	Involved in the study
☐	☒ Antibodies
☐	☒ Eukaryotic cell lines
☒	☐ Palaeontology and archaeology
☐	☒ Animals and other organisms
☒	☐ Clinical data
☒	☐ Dual use research of concern
☒	☐ Plants

Methods

n/a	Involved in the study
☒	☐ ChIP-seq
☐	☒ Flow cytometry
☒	☐ MRI-based neuroimaging

Antibodies

Antibodies used

Mouse anti-human CD19-PE-Cy7 (1:100; BD Biosciences Pharmingen™, San Diego, CA, USA; Mat. No. 557835; Clone: SJ25C1; Lot#: 8194923)
Donkey anti-human IgM-PE (1:50; Jackson ImmunoResearch Laboratories Inc., West Grove PA, USA; Code No., 709-116-073; polyclonal; Lot#: 132757)
Goat anti-human IgA-APC (1:20; Jackson ImmunoResearch Laboratories Inc., West Grove, PA, USA; Code No., 109-135-011; polyclonal; Lot#: 131656)
Mouse anti-human IgD-FITC (1:20; BD Biosciences Pharmingen™, San Diego, CA, USA; Mat. No., 555778; Clone: G155-178; Lot #: 7299866)
Donkey anti-human IgG-HRP (1:5,000; Jackson ImmunoResearch Laboratories Inc., West Grove, PA, USA; Cat. No., 709-035-149; Polyclonal; Lot #153143)
Goat anti-mouse IgG-HRP (1:5,000, Sigma-Aldrich, St. Louis, MO, USA; Code No.: 115-035-062; Lot #: 134308)
Mouse anti-human IgG3-HRP (1:1,000; Invitrogen, Thermo Fisher Scientific, Inc., Rockford, IL, USA; Ref#: 05-3620; Lot #: WF318915)
Goat anti-human F(ab)-HRP (1:40,000; Jackson ImmunoResearch Laboratories Inc., West Grove, PA, USA; Source#: 147767; Batch#: 157201)
Serotype-specific anti-DENV VLP, anti-JEV VLP, and anti-ZIKV VLP sera or MHIAF were either in-house prepared or kindly provided by Dr. G.-J. Chang (recently retired from Division of Vector-borne Diseases, Centers for Disease Control and Prevention, DVBD-CDC, Fort Collins, CO, USA).
Polyclonal and purified total human IgGs were isolated from human volunteers, PV10 and TW2.
Pan-flavivirus anti-E murine MAb FL0231 and anti- NS1 murine MAb mFL0221 were kind gifts from Dr. L.-K. Chen (Tzu Chi University Hospital, Hualien, Taiwan)

Validation

Antibodies were chosen based on the validation statements for species (human) and application (FACS or ELISA) on the manufacturer's website.
The serotype-specific anti-DENV VLP, anti-JEV VLP, and anti-ZIKV VLP sera or MHIAF were in-house produced or donated, and were validated by various antigen-specific ELISAs. These antibodies have also been reported in different research articles such as Galula et al., J. Virol. (2014) 88, 10813–10830 (PMID: 25008922), Shen et al., Elife (2018) 7, 1–24 (PMID: 30334522), Chao et al., J Clin Microbiol (2019) 57(3):e01506-18 (PMID: 30541932), and Galula et al., Diagnostics (2021) 11, 741 (PMID: 33919324).
The polyclonal and purified total human IgGs were purified from the whole blood of donors TW2 and PV10 after obtaining informed consent (under Kaohsiung Medical University IRB Approval (IRB No. KMUHIRB-E (II)-20180092). Purified total IgGs were validated by visualization in SDS-PAGE with target band at ~150 kDa, and for specificity using flavivirus-specific VLP binding ELISAs.
The purified pan-flavivirus anti-E murine MAb FL0231 and anti-NS1 murine MAb mFL0221 were validated in SDS-PAGE, identified as a band at ~150kDa, and has been reported to specifically recognize the E and NS1 flavivirus proteins. These antibodies have been used and reported in our previous publication such as Galula et al., Diagnostics (2021) 11, 741 (PMID: 33919324).

Eukaryotic cell lines

Policy information about cell lines and Sex and Gender in Research

Cell line source(s)

Vero (CRL 1587; ATCC, Manassas, VA, USA) were initially provided by Dr. G.-J. Chang (recently retired from Division of Vector-borne Diseases, Centers for Disease Control and Prevention, DVBD-CDC, Fort Collins, CO, USA) and maintained in DYC's lab with detailed passage records.
HEK293T (CRL-3216; ATTC, Manassas, VA, USA) were purchased from ATCC and maintained with detailed passage records.
Both Vero and HEK29T cells have been routinely used by our lab and reported in previous works Galula et al., J. Virol. (2014) 88, 10813–10830 (PMID: 25008922), Shen et al., Elife (2018) 7, 1–24 (PMID: 30334522), and Galula et al., Diagnostics (2021) 11, 741 (PMID: 33919324).
Human PBMCs were isolated using the density-gradient centrifugation from donor KH1891 after informed consent was obtained and after approval from Kaohsiung Medical University IRB Approval (IRB No. KMUHIRB-E (II)-20180092).
EL4-B5 thymoma cells were provided by Dr. Cheng-I Wang, one of the authors, and were propagated and maintained using standard cell culture protocols with detailed passage history.
The NS1/1-Ag4-1 mouse myeloma cells (TIB-18; ATCC, Manassas, VA, USA) were purchased from ATCC, maintained and propagated using standard cell culture procedures with detailed passage history.

Authentication

No authentication was performed in commercially purchased cells such as HEK293T, Vero cells, and NS1/1-Ag4-1 mouse myeloma cells.

Mycoplasma contamination

All the cells used were negative for mycoplasma and cell cultures were checked regularly for mycoplasma contamination by following commercial protocol (MycoStrip™, Cat. No.: Rep-Mys-50, InvivoGen, Hong Kong).

Commonly misidentified lines (See ICLAC register)	No misidentified reports were found for the cell lines used in this study.

Animals and other research organisms

Policy information about studies involving animals; ARRIVE guidelines recommended for reporting animal research, and Sex and Gender in Research

Laboratory animals	Mice used in this study were 3-week-old BALB/c (Jy) female mice and were purchased from NARLAbs, National Animal Laboratory Center, Taipei City, Taiwan. Mice were housed in the specific pathogen-free animal house at the National Chung Hsing University in accordance with the guidelines for the care and use of animals as approved by the Institutional Animal Care and Use Committee (IACUC) with approval number: 106-101R2.
Wild animals	The study didn't involve wild animals.
Reporting on sex	This findings in the study are only applied to female mice since gender difference is not the concern in this study.
Field-collected samples	The study did not involve samples collected from the field.
Ethics oversight	Institutional Animal Care and Use Committee (IACUC) of the National Chung Hsing University (Approval Number: 106-101R2)

Note that full information on the approval of the study protocol must also be provided in the manuscript.

Flow Cytometry

Plots

Confirm that:

☒ The axis labels state the marker and fluorochrome used (e.g. CD4-FITC).

☒ The axis scales are clearly visible. Include numbers along axes only for bottom left plot of group (a 'group' is an analysis of identical markers).

☒ All plots are contour plots with outliers or pseudocolor plots.

☒ A numerical value for number of cells or percentage (with statistics) is provided.

Methodology

Sample preparation	Under sterile conditions, human peripheral blood mononuclear cells (PBMCs) were freshly isolated using density gradient centrifugation with Ficoll-Paque PLUS (GE Healthcare, Uppsala, SWE), washed with PBS to remove non-target cell types, and resuspended in RPMI media for 24 hrs at 4°C. On the day of sorting, the PBMCs were stained on ice with fluorophore conjugated anti-human antibodies against CD19 (BD Biosciences), IgM (Jackson ImmunoResearch Laboratories), IgA (Jackson ImmunoResearch Laboratories) and IgD (BD Pharmingen). These antibodies were directly coupled to fluorescein isothiocyanate (FITC), allophycocyanin (APC), phycoerythrin (PE) and tandem fluorochrome phycoerythrin-cyanine 7 (PE-Cy7) to distinguish among B cell populations. A staining mastermix composed of the four fluorophore-conjugated antibodies was prepared, spun down at 3,000xg for 20 minutes at 4°C. 10-50 x 106 PBMCs were added to a pre-warmed complete Iscove's modified Dulbecco's medium (IMDM, Gibco) containing 10% heat-inactivated fetal bovine serum with 15uL benzonase and spun down at 335g at 4°C for 10 minutes. Pelleted cells were resuspended in 1mL of PBS-1% (wt/vol) BSA supplemented with 1% HEPES, which served as the sorting buffer. Fifty microliters (50 uL) of cell aliquot was transferred to each of the five new 15-mL conical tubes for compensation in flow cytometry. The cells in all the tubes were pelleted at 335g at 4°C for 10 minutes, following the protocol by Huang et al., Nature Protocols (2013), 8 (10), 1907-1915 (PMID: 24030440).
Instrument	BD FACSMelody™ Cell sorter (Becton, Dickinson, and Company)
Software	BD FACSChorus™ software
Cell population abundance	The abundance of the post-sort fractions were higher than 97%.
Gating strategy	Cells were loaded onto the cytometer and gated on the CD19+IgM-IgA-IgD- B cells as shown in the Supplementary Figures. Gating was initially set according to cell size and granularity or 'events' in order to minimize the presence of doublets. Target monocytes were defined by the cell size or length parameter, which is indicated by the forward scatter (FSC) signal while that of granularity was shown by the side scatter (SSC) signal. Doublets were excluded using forward light-scattering gating followed by gating on lymphocytes based on FSC/SSC. To determine whether potentially contaminating cell types were effectively gated-out, different cells populations with antibodies against B cells (CD19), IgM, IgA and IgD were first identified.

☒ Tick this box to confirm that a figure exemplifying the gating strategy is provided in the Supplementary Information.
